# First Report of *Diplodia quercivora* and *Neofusicoccum vitifusiforme* Associated with Cankers and Necrosis of Holm Oak (*Quercus ilex*) in Declining Stands in Southern Italy

**DOI:** 10.3390/jof10010035

**Published:** 2024-01-03

**Authors:** Carmine Del Grosso, Davide Palmieri, Lucia Marchese, Luigi Melissano, Giuseppe Lima

**Affiliations:** 1 Department of Agricultural, Environmental and Food Sciences, University of Molise, 86100 Campobasso, Italy; davide.palmieri@unimol.it (D.P.); l.marchese@studenti.unimol.it (L.M.); 2 Institute for Sustainable Plant Protection, National Research Council (CNR), 70126 Bari, Italy; 3 Department of Agriculture, Environment and Rural Development, Sustainable Management and Protection of Natural and Forest Resources, Apulia Region, 70100 Bari, Italy; l.melissano@regione.puglia.it

**Keywords:** forest decline, *Diplodia corticola*, *Botryosphaeriaceae*, emerging plant diseases, fungal pathogens, endophytic fungi, biotic and abiotic stress

## Abstract

The emergence of new plant diseases is an increasingly important concern. Climate change is likely to be among the factors causing most of the emerging diseases endangering forest and tree heritage around the world. Such diseases may be caused by latent pathogens or microorganisms cryptically associated with plants. The shift from a non-pathogenic to a pathogenic stage may depend on physiological alterations of the host, environmental changes, and/or stress factors. In some woods of the Salento Peninsula (Apulia Region, Italy), sudden declines of holm oak plants (*Quercus ilex* L.) have been observed since 2016. The morphological and molecular characterization of representative fungal isolates associated with cankers and necrosis in declining plants indicated that these isolates belong to the *Botryosphaeriaceae* family, and the most frequent species were *Diplodia corticola* and *Diplodia quercivora*, followed by *Neofusicoccum vitifusiforme*. In artificially inoculated young holm oak plants, both *D. corticola* and *D. quercivora* species produced intense and severe subcortical and leaf margin necrosis. *N. vitifusiforme*, although less aggressive, induced the same symptoms. Our research, in addition to confirming the involvement of *D. corticola* in olm oak decline, represents the first report of *D. quercivora* as a new pathogen of *Q. ilex* in Italy. Furthermore, to the best of our knowledge, we also found *N. vitifusiforme* as a new pathogen of *Q. ilex*.

## 1. Introduction

Due to several factors, Emerging Infectious Diseases (EIDs) have been increasingly threatening trees in agroforestry and urban ecosystems [1], thus posing a growing worldwide problem over the last few decades [2,3]. Over the centuries, the main cause of the spread of EIDs has been the introduction of alien pathogens into new geographic areas, mainly due to the increase in international trade [4]. However, other factors, such as climate change, also play a key role in the evolutionary and adaptation processes involved in the expansion of pathogens toward new favorable areas [5].

Globalization has certainly favored the spread of previously geographically isolated plant pathogens [4], also favoring their hybridization with related species (and/or newly introduced species) in new environments with different and/or wider host ranges than the parental species [6], i.e., by the acquisition of novel traits of virulence from other species through horizontal gene transfer [7,8]. EIDs can be caused by a multitude of factors, among which the most relevant are: (i) the emergence of new virulent strains or species); (ii) new vector–pathogen associations; (iii) the introduction of new crops and cultivation practices (e.g., the global use of intensively managed forest plantations of non-native species or of a small number of clones of the same species); (iv) latent or cryptic plant pathogens [5]. Cryptic plant pathogens are microorganisms that share a strong resemblance or are morphologically indistinguishable from recognized widespread non-pathogenic species that possess hidden characteristics by which they can abruptly cause severe and unexpected diseases [9,10], thus evolving into new, more aggressive biotypes [2,11,12]. 

There are many examples of endophytic fungi commonly isolated from asymptomatic plants that, under changed environmental conditions and/or various stressors altering the host physiology, can evolve as necrotrophic pathogens [13,14,15]. Emerging pathogens characterized by a considerable latency stage are usually difficult to detect [16,17]. Fungal trunk pathogens are classic examples of a very diverse group of microorganisms including well-studied endophytes and latent pathogens of woody plants that typically cause diseases associated with some type of stress [18]. The species belonging to the *Botryosphaeriaceae* family are among the most aggressive pathogens commonly described as endophytic fungi. They are ubiquitous and occur on a variety of plant hosts, causing dieback and canker diseases [19]. 

The aim of the present study is to identify the causal agents of the sudden decline of holm oak plants (*Q. ilex*), which was first observed in 2016 in some woods of the Salento Peninsula in the Apulia Region of Italy. Following inspections in woods affected by canopy decline, cankers, and subcortical necrosis, sampling and isolations were carried out from symptomatic holm oak branches and trunks in a variety of locations in the Salento Peninsula. Fungal isolates most frequently associated with symptomatic plants were morphologically and molecularly characterized and used in pathogenicity tests on artificially infected young holm oak plants to demonstrate Koch’s postulates.

## 2. Materials and Methods

### 2.1. Visual Inspection of Trees, Sampling, and Isolation

**Figure 1:**
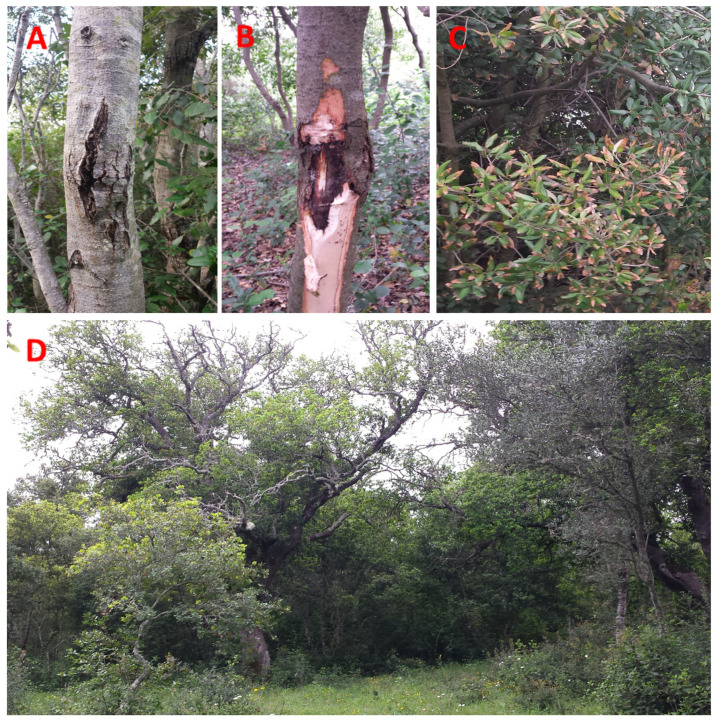
Representative symptoms observed in the analyzed declining plants in the holm oak forests. (**A**–**C**) Cankers and subcortical necrosis; (**D**) stunting and decline of young holm oak plants.

Field surveys were carried out in the spring–summer period of the years 2016, 2017, and 2022 in seven woodlands of the Salento Peninsula (Province of Lecce, Apulia Region, Italy) (Table 1) affected by canopy decline, branch and trunk cankers, and subcortical necrosis (Figure 1). 

Meteorological data of the area including the seven monitored woodlands in the province of Lecce were taken from the website https://www.ilmeteo.it/portale/archivio-meteo/Lecce (accessed on 10 October 2023). The acquired meteorological data, encompassing mean seasonal temperatures and total precipitation of the year 2022, served as a basic reference for the environmental conditions observed during the investigation period. 

These data revealed the following mean seasonal temperatures and total precipitation: (i) Spring: Mean temperature (MT) 13.7 °C, Precipitation (P) 22 mm; (ii) Summer: MT 26.7 °C, P 18.7 mm; (iii) Autumn: MT 17.9 °C, P 75 mm; (iv) Winter: MT 9.5 °C, P 58 mm.

The woodlands examined in this study represent the typical natural stands of holm oak forests of southern Italy and they are characterized by a rich and diverse ecosystem with a prevalence of holm oak (*Q. ilex*), and in some instances, kermes oak (*Quercus coccifera*) as woody tree species. The structure of these woodlands is complex, with trees of different ages and sizes forming a dense underbrush. They come mainly from old reforestation or from what remains of ancient forests and/or green parks. They are forests not primarily intended for forestry use, but rather aimed at landscape preservation, tourism, and recreation. In some cases, they also serve to meet the local population’s need for firewood or, rarely, for the use of high-quality wood by local artisans. High scrubland, in association with oak, comprises strawberry trees (*Arbutus unedo*), buckthorns (*Rhamnus alaternus*), and tree heaths (*Erica arborea*). Low scrubland differs from the high scrubland due to the lower frequency of species typical of the holm oak forests, with the disappearance of those more suited to shaded environments, and the presence of species typical of the garrigue. In southern Salento, low scrubland is characterized by the prevalent presence of spiny broom (*Calicotome infesta*) and myrtle (*Myrtus communis*), accompanied by flax-leaved daphne (*Daphne gnidium*), mastic trees (*Pistacia lentiscus*), wild asparagus (*Asparagus acutifolius*), and clematis (*Clematis* spp.). 

Each woodland was divided into 400-square-meter grids, and three sampling areas were randomly selected in each woodland. Within each sampling area, two symptomatic holm oak trees were selected and representative sections of symptomatic branches or trunks were taken from each plant and used for fungal isolation. The samples were superficially sterilized by immersing them for 1 min in a water solution of sodium hypochlorite with 2% of active chlorine, then samples were washed in 70% ethanol for 30 sec, rinsed in sterile distilled water 3 times, and placed to dry in aseptic conditions. The bark was removed with a sterile scalpel from the sterilized section showing discoloration and necrosis of the subcortical layer, also in depth. From the margin of symptomatic areas, five small pieces of inner bark and xylem tissues (about 3–4 mm) were taken from each sample and plated in Petri dishes on potato dextrose agar (PDA) enriched with antibiotics (streptomycin 250 µg/mL and ampicillin 100 µg/mL). The plates were incubated at 24 ± 2 °C for 5 days in the dark. All growing colonies were transferred onto fresh PDA plates without antibiotics. To purify fungal colonies, the monoconidial and/or monohyphal culture method was used. Briefly, a small amount of conidia and/or mycelium biomass was scraped from 7-day-old fungal colonies and suspended in 10 mL of distilled water. The solution was vortexed for 30 sec at high speed and filtered through four layers of sterile gauze, and a drop (10 µL) of conidial or mycelium fragment suspension was inoculated on Water-Agar (WA) and incubated at 24 ± 2 °C for 24–48 h in the dark. The axenic fungal colonies were obtained by taking single germinated conidia or a fragment of germinated hyphal tips, transferred onto PDA, and incubated at 24 ± 2 °C in the dark for 1 week. Among the pure isolated colonies, based on their macroscopic and microscopic characteristics, three of the most representative fungal colonies were subjected to further taxonomic investigations.

### 2.2. Morphological Identification of the Fungal Isolates

The fungal isolates were subjected to further observation of the main morpho-cultural characteristics (growth rate, color, structure of the mycelium, production of diffusible pigments) at different temperatures and on different substrates in accordance with the reference literature [20,21,22,23]. 

Three strains were selected as the most representative morphotypes based on macroscopic and microscopic observations. These strains were unable to produce spores during conventional cultivation on PDA, even after many days of incubation. Therefore, agar plugs with mycelium were transferred onto agarized media poor in nutrients (10-times diluted PDA) and incubated at 24 ± 2 °C, and, after 5 days, the fungal colonies were exposed to UV light (320 nm of wavelength) for 1 min per day. Pycnidia and conidia were sampled from each plate and observed under a light microscope, analyzing the shape and dimensions of each structure. Pictures were taken using an Olympus microscope (mod. BH-2, Tokyo, Japan) and a Zeiss stereomicroscope (Stemi 2000-C, Jena, Germany). The dimensions of the conidia were assessed by using the measuring eyepiece of the microscope with a magnification of 400–1000× and measuring at least 10 conidia per fungal isolate.

### 2.3. Pathogenicity Tests

To evaluate the pathogenicity and virulence of the three selected fungal isolates (one representative fungal isolate of each of the three most frequently isolated morphotypes), preliminary biological tests were carried out on 2-year-old holm oaks (*Q. ilex*) grown in plastic pots filled with a soil–peat mixture (2:1). To promote the infectious process and the subsequent development of symptoms, for each fungal isolate, nine plants per isolate (3 × 9 plants) and nine control plants were stressed (root asphyxia) by immersing the pots with the root system in water for 7 days, followed by a period of 5 days of dryness in a screen house under natural light condition (temperature 28 °C and humidity < 45%). The inoculation area (about 30 cm above the root collar) was surface disinfected on the bark with 70% ethanol, then a portion of the bark was removed with a sterile cork borer (Ø 6 mm), and a disk of fungal mycelium (Ø 6 mm), taken from the margin of a 5-day-old PDA culture, was placed in contact with the wood. The inoculation point, including the agar disk, was covered with cotton wool soaked in sterile distilled water, and then with a layer of parafilm, to preserve moisture. As a negative control, the plants were subjected to the same treatment, but using an agar disk of the same medium without fungal mycelium. The surveys were carried out 40 days after inoculation at the appearance of slight foliar symptoms on the inoculated plants. To this end, the inoculation points were revealed by removing the parafilm and cotton wool. The cortex was removed with a sharp blade in order to reveal any subcortical discoloration/necrosis and its length was accurately measured and analyzed. The same protocols reported in the previous two paragraphs were followed to isolate the fungal pathogens from the symptomatic tissues and to characterize the selected isolate. 

### 2.4. Molecular Characterization of the Isolates

The genomic DNA was isolated from the fungal mycelium (200 mg) collected from 7-day-old inoculated PDA plates. The mycelium was transferred into 1.5 mL centrifuge tubes with 500 μL of extraction buffer (0.1 M NaCl, 0.5 M Tris-HCI, pH 8.0, 5% sodium dodecyl sulfate) and approximately 0.2 g of glass beads for cell lysis. The tubes were vortexed for 10 min at maximum intensity, placed at −30 °C for 15 min, and centrifuged for 10 min at 10,000 RPM. An equal volume of phenol–chloroform–isoamyl alcohol (25:24:1) was added to 200 μL of the collected supernatant and vortexed and centrifuged for 5 min at 10,000 RPM. Next, 250 μL of the aqueous phase was taken and 625 μL of isopropanol was added to the tube. The tubes were then kept at 2 °C for 1 h and centrifuged for 10 min at 14,000 RPM. The isopropanol was discarded, and the pellet was washed with 70% ethanol and centrifuged for 10 min at 14,000 RPM. The supernatant was discarded, and the DNA pellets were left to dry at room temperature for 1 h and finally re-suspended in 50 μL of TE buffer at pH 8 and stored at 4 °C or at −20 °C. Through PCR analysis, the ITS and TEF-1α regions were amplified by using the pairs of primers ITS1 and ITS4 [24] and EF1-688F and EF1-986R [25], respectively. The PCR reaction was carried out in a total volume of 25 μL containing 6.5 μL of H_2_O; 2 μL of each primer, 12.5 μL of PCR Master Mix (Promega, Madison, WI, USA), and 2 μL of DNA template.

The amplifications were performed using a T Gradient Thermal Cycler mod. 512 (Techne, Burlington, NJ, USA) under the following conditions: initial denaturation of 2 min at 94 °C, followed by 40 cycles of denaturation of 30 s at 94 °C, annealing of 30 s at 55 °C, and extension of 1 min at 72 °C with a final extension of 5 min at 72 °C.

The PCR products were loaded onto an agarose gel (50 mL TAE 1×, 0.4 g agarose, with the addition of 2 μL of ethidium bromide) run at 80 V for 1 h and visualized using the Gel Doc 2000 transilluminator (Bio-Rad, Hercules, CA, USA).

All the obtained ITS and TEF-α amplicons were first purified using the NucleoSpin extract II purification kit (Macherey-Nagel, Düren, Germany) and sent for sanger sequencing at Eurofins Genomics. The nucleotide sequence of each isolate was then compared with those present in the GenBank database using the “Nucleotide BLAST” tool (https://blast.ncbi.nlm.nih.gov/ (accessed on 27 April 2023) in order to verify the similarity with sequences already present in the database (similarity percentages varying from 99 to 100%).

For the phylogenetic analysis, merged ITS and TEF-1 α nucleotide sequences were aligned, corrected, and analyzed using Molecular Evolutionary Genetics Analysis (MEGA X, v. 11) software together with reference sequences downloaded from GenBank (www.ncbi.nlm.gov (accessed on 27 April 2023)), utilizing a strain of *Lasiodiplodia mediterranea* and a strain of *Xylaria hypoxylon* as outgroups (Table 2). Reference taxa were selected based on sequence blast results analysis, and all reference sequences were randomly selected from the NCBI Taxonomy Browser (https://www.ncbi.nlm.nih.gov/Taxonomy/Browser/wwwtax.cgi (accessed on 27 April 2023), selecting only species isolated from agricultural and forestry plants according to Batista and colleagues’ (2021) database [26]. After the alignment and the exclusion of incomplete portions from both ends, the most suitable models for dendrogram creation and phylogenetic analysis were selected by using the function “Find the Best DNA/Protein Models” (ML) of the MEGA X v. 11 software, thus determining the most appropriate evolutionary model for the purposes of the analysis. The maximum likelihood model (MLM) was used to evaluate the robustness of the tree obtained by setting the bootstrap number to 1000 replicates using the parameter model Tamura 3 and Gamma distributed function, with 5 discrete Gamma Categories, and gaps/missing data treated as complete deletions.

### 2.5. Statistical Analysis

Data from pathogenicity tests were analyzed with nonparametric tests comparing the cumulative distributions of the dataset; significant differences were determined using the two-tailed Kolmogorov–Smirnov test with a 95% confidence level (*p* < 0.05). 

Statistical analyses were performed with GraphPad Prism version 8.0.0 for Windows, GraphPad Software, San Diego, CA, USA.

## 3. Results

### 3.1. Morphological Characterization of the Fungal Isolates

**Figure 2:**
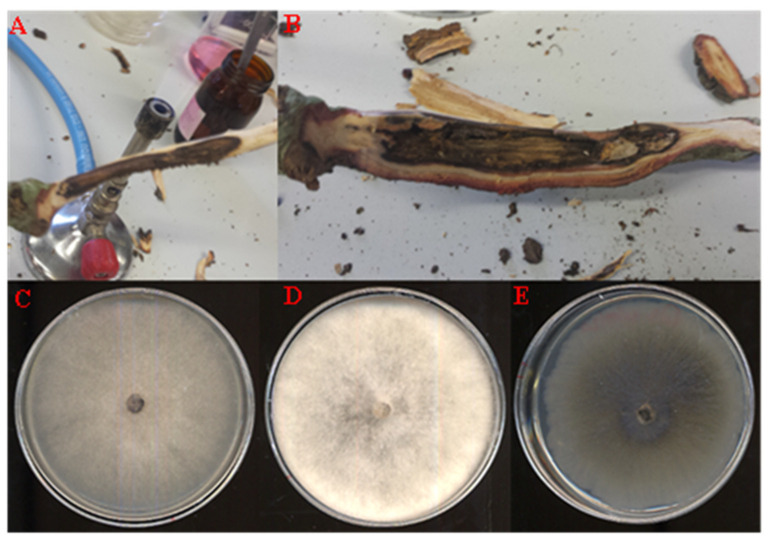
(**A**,**B**): Representative symptomatic oak holm samples used for the fungal isolation. (**C**–**E**): Axenic culture obtained after 7 days of incubation on PDA in Petri dishes (Ø 90 mm) for the most frequent fungal isolates QLE1 (**C**), QLE2 (**D**), and QLE3 (**E**).

Isolations carried out from symptomatic oak tissues (Figure 2A,B) allowed us to collect and store a variety of fungal isolates in our fungal collection. Among these, around 54% of the isolates were categorized as *Botryosphaeriaceae* (114 fungal colonies on a total of 210 segments incubated) and were grouped and divided into five different morphotypes by macroscopic and microscopic observations. A preliminary PCR and sequence analysis of the ITS region from randomly selected fungal isolates for each morphotype enabled us to identify different species of *Botryosphaeriaceae*, with a high frequency of isolation (isolation rate %, IR = Ni/Nt × 100, in which Ni = number of colonies isolated for each morphotype and Nt = total number of segments incubated). A high isolation frequency was found for *Diplodia corticola, D. quercivora,* and *N. vitifusiforme* morphotypes, with IR values of 20.5%, 16.7%, and 5.2%, respectively, while a low frequency was shown for *D. mutila* (IR = 0.5%) and *D. gallae* (IR = 2.4%). The remaining 9% of *Botryosphaeriaceae* isolates have not been classified, and about 45% of the segments were contaminated by *Penicillium* spp., *Rhizopus* spp., and other contaminant microorganisms. Among the most frequently found fungal colonies, three representative isolates were selected for this study. These isolates were coded as QLE1 (representing the *D. corticola* morphotype), QLE2 (representing the *D. quercivora* morphotype), and QLE3 (representing the *N. vitifusiforme* morphotype). 

Both the isolates QLE1 and QLE2 developed a cottony mycelium with a color ranging from white to dark grey, which quickly colonized the entire Petri dish (Figure 2C,D). After 14 days of incubation, isolate QLE1 produced numerous and mainly stromatic pycnidia, while isolate QLE2 produced a few pycnidia, in most cases quite spaced out and embedded between the growth substrate and the mycelium.

Isolate QLE3 produced colonies with smooth regular margins, white on the surface and greenish olive underneath, tending to darken toward the central point of the colony. No reproductive structures were observed for this isolate on PDA (Figure 2E).

**Figure 3:**
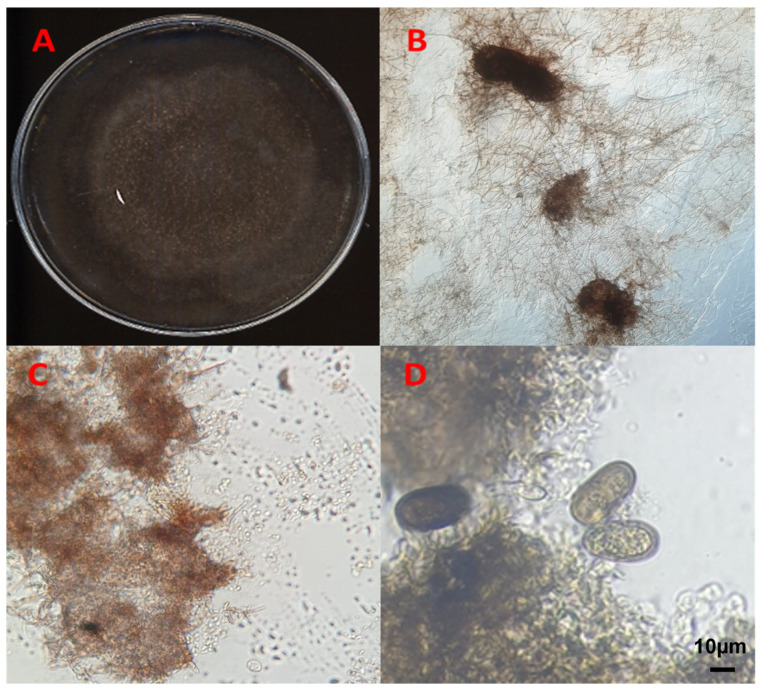
*Diplodia corticola* isolate QLE1: (**A**) 14-day PDA culture; (**B**) 40× light microscope pycnidia; (**C**) 100× light microscope conidia leakage; (**D**) conidia under light microscopy at 800×.

**Figure 4:**
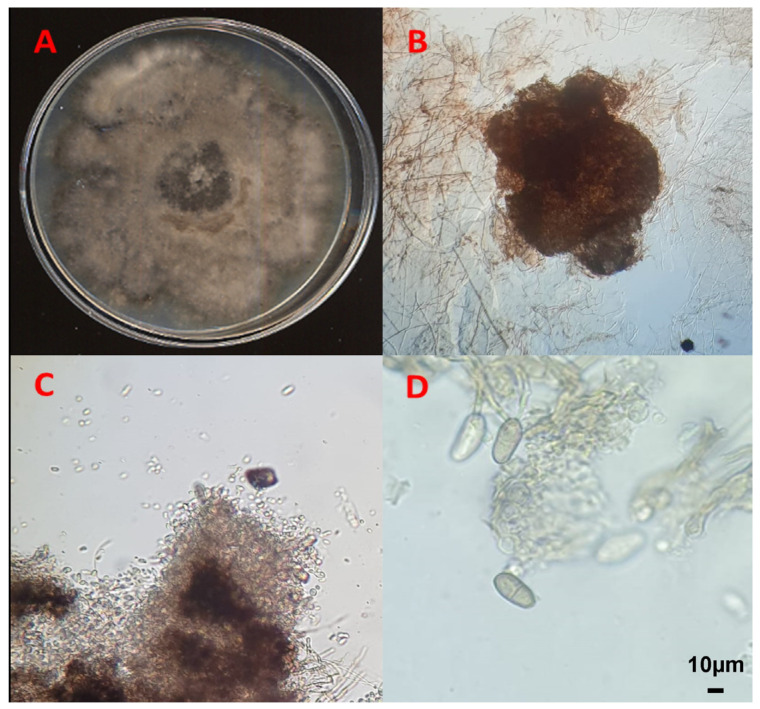
*Diplodia quercivora* isolate QLE2: (**A**) 14-day PDA culture; (**B**) 20× light microscope pycnidia; (**C**) 100× light microscope conidia leakage; (**D**) conidia under light microscopy at 600×.

The fungal sporulation assay conducted on 10-times diluted PDA under UV light stress gave positive results for isolates QLE1 and QLE2, where it was possible to observe the entire reproductive structures after 7 and 14 days, respectively, when they developed pycnidia at the edges of the plates embedded between the agar and fungal mycelium (Figure 3 and Figure 4).

**Figure 5:**
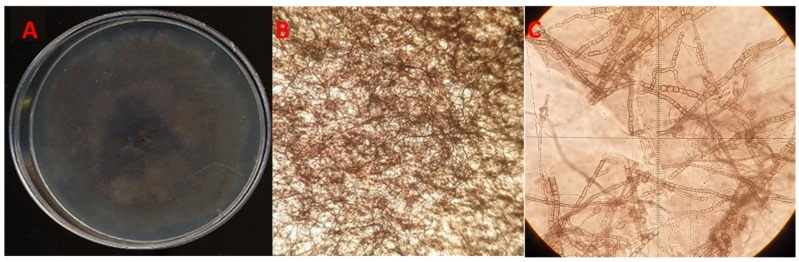
*Neofusicoccum vitifusiforme* isolate QLE3: (**A**) 14-day PDA culture; (**B**) mycelium under light microscope at 40×; (**C**) arthroconidia under light microscope at 400×.

It was not possible to obtain the production of spores for isolate QLE3 with any of the techniques used. However, a careful microscopic observation of the samples allowed us to observe arthroconidia, consisting of agamic fungal structures (thallic conidia), which derived from the fragmentation of a mycelial hypha in the hyphal septa (Figure 5C).

### 3.2. Pathogenicity Test

**Figure 6:**
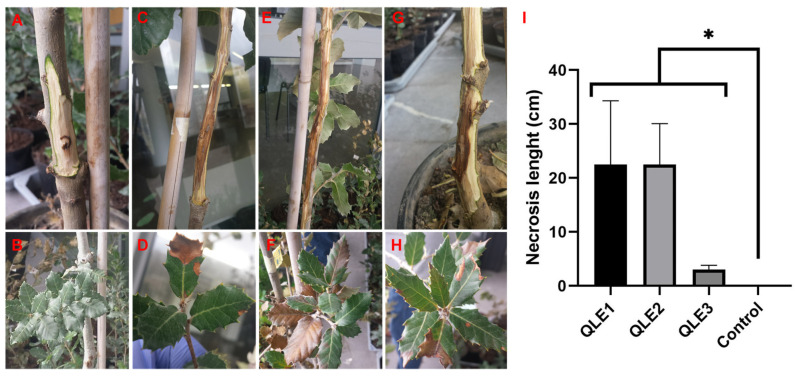
Pathogenicity assays on young holm oak plants in controlled conditions. Representative images of symptoms, wood necrosis, and discoloration extension in plants used for the pathogenicity assay at 40 days post inoculation (dpi). (**A**,**B**) Non-inoculated control; (**C**,**D**) plant inoculated with isolate QLE1; (**E**,**F**) plant inoculated with isolate QLE2; (**G**,**H**) plant inoculated with isolate QLE3. In the graph (**I**), the average and standard deviation of the length of the subcortical necrosis caused by the three strains on inoculated young holm oak at 40 dpi are reported. A nonparametric test comparing the cumulative distributions of the dataset was used, and significant differences were determined by the two-tailed Kolmogorov–Smirnov test with 95% confidence level (* *p* < 0.05).

Preliminary pathogenicity tests showed that the three representative strains isolated from symptomatic holm oak (*Q. ilex*) were able to develop the same symptoms observed in the field on artificially inoculated plants. In particular, isolates QLE1 (Figure 6C,D) and QLE2 (Figure 6E,F) proved to be highly aggressive, producing extended subcortical necrosis of 22.5 cm (SD = ±11.8) and 22.5 cm (SD = ±7.6), respectively, and leaf marginal necrosis at 40 days post inoculation. Conversely, isolate QLE3 was less aggressive, but capable of causing subcortical browning necrosis of 3.0 cm (SD = ±0.8), and leaf marginal necrosis (Figure 6G,H). No necrosis and symptoms were observed in control plants (Figure 6A,B).

### 3.3. Molecular Identification of the Fungal Isolates

The molecular analyses highlighted that the three fungal isolates belong to the *Botryosphaeriaceae* family. Isolate QLE1 was identified as *Diplodia corticola* (ITS: Percent Identity 100%; TEF-1α: Percent Identity 100%), a known woody plant pathogen of oaks and other forest species. Isolate QLE2, belonging to the same genus, was identified as *D. quercivora* (ITS: Percent Identity 100%; TEF-1α: Percent Identity 99.58%), also a known woody plant pathogen of various woody plant species, and in particular, oaks. Finally, the last isolate QLE3 was classified as *Neofusicoccum vitifusiforme* (ITS: Percent Identity 99.8%; TEF-1α: Percent Identity 100%). This fungal species, unlike the previous ones, is a known pathogen in the agricultural environment, mainly on grapevines, but it has never been reported as an oak pathogen in Europe.

**Figure 7:**
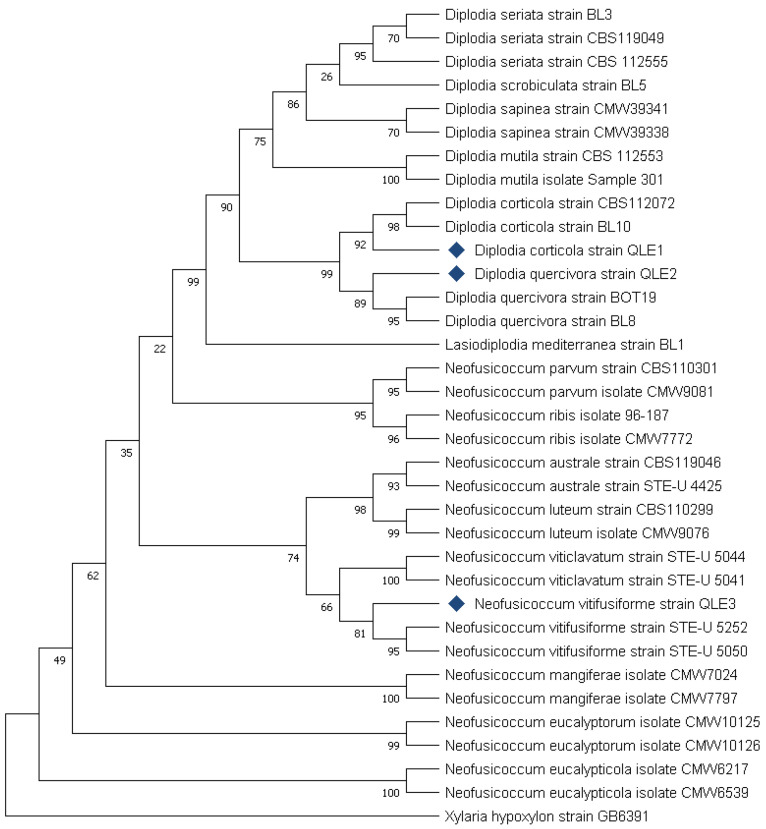
Phylogenetic analysis conducted using the ITS and TEF-1 α sequences of the three isolates used in this study. Species isolated from agricultural and forestry plants have been included as reference sequences. The two “outgroup” strains are represented by *Lasiodiplodia mediterranea* strain BL1 and *Xylaria hypoxylon* strain GB6391. The “bootstrap” values represent the result of 1000 replicates.

The phylogenetic analysis of the three *Botryosphaeriaceae* isolates confirmed the identification obtained by aligning the nucleotide sequences. Based on the characteristics of the ITS and the TEF-1α regions, the phylogenetic analysis enabled us to identify the isolates as *D. corticola* and *D. quercivora*, respectively, with positive confidence of the results (Bootstrap values 99 and 89, respectively) (Figure 7).

In the case of *N. vitifusiforme* (isolate QLE3), despite the fact that in the phylogenetic analysis, it grouped with reference isolates of the same species, bootstrap values did not confirm the reliability of the results (Bootstrap value 81). However, the isolate was clearly distinct from other species of the same genus (Figure 7).

## 4. Discussion

Emerging infectious plant diseases have complex causes related to the current dramatic global-scale changes in climate, trade, and human behavior [5]. Consider that biological invasion, a generally human-mediated event linked to global trade and climate change, provides multiple opportunities for the introduction and spread of alien pathogens [4]. In the last century alone, the range of the distribution of many plant pathogens has extended beyond their natural limits, causing enormous ecosystem disturbances and serious socio-economic impacts. The factors determining the emergence of diseases are often multifaceted and their effects should generally be considered and studied in relation to their possible multiple interactions [27].

The emergence of new diseases, either latent or cryptic, or caused by fungal pathogens that colonize new areas due to climate change, is a recent phenomenon of particular concern. In Italy, as well as in the rest of Europe, increasingly frequent reports of tree decline in oak forests have been recorded and a range of oak pathogens, for example, *Armillaria* spp., *Cephalosporium* spp., *Cladosporium* spp., *Cylindrocarpon* spp., *Diplodia* spp., *Biscogniauxia mediterranea* (*Hypoxylon mediterraneum*), *Phoma cavae, Phomopsis quercina, Sporotrix* spp., and *Phytophthora* spp. have been found to be associated with this decline [27,28,29,30,31,32,33,34]. It is not yet clear whether the disease development in many of these cases is primarily caused by emerging pathogens, as, for example, found in Italy for *Diplodia corticola* and *Phytophthora cinnamomi* on declining holm oak in Caprera Island (Sardinia) [28] and in the Salento Peninsula (Apulia) [35], or whether tree decline is due to physiological stresses caused by climate change, as in the case of the Lucanian Apennine (Basilicata Region) in Italy [36]. In most cases, the increase in these reports derives from the negative interaction of both factors, phytopathogens and environmental stresses [5]. 

In this work, following recent reports of holm oak (*Q. ilex*) declining in woods of the Salento Peninsula (Apulia Region of Italy), we carried out several surveys, samplings, and fungal isolations from seven representative woodlands of the area of interest. From the collected samples, fungal colonies belonging to the *Botryosphaeriaceae* family were the most frequently isolated.

Pathogenicity tests have shown that the three fungal strains isolated from holm oak (*Q. ilex*) are able to generate severe disease symptoms on artificially inoculated healthy plants. Furthermore, in line with Koch’s postulates, the fungal strains were re-isolated at the conclusion of the tests and their morphological characteristics coincided with those of the initially inoculated strains.

*Diplodia corticola* is a fungal species already known as a dangerous woody plant pathogen of oaks and other forest tree species [21,28] and described in Italy as a causal agent of the decay of holm oak woods [28]. *D. quercivora* is recognized as a pathogen of *Quercus* spp. in other countries [29,30,31,37]. However, our study has shown that this species is also associated as a pathogen with holm oak in Italy. 

Regarding *N. vitifusiforme*, it was reported to be a grapevine pathogen in Italy [38] and described in association with other *Botryosphaeriaceae* on *Quercus suber* declining forests in Algeria [32]. To the best of our knowledge, our study represents the first report of this species as a potential oak pathogen in Europe. Further investigations are in progress to better understand and evaluate its pathogenicity on holm oak. Regarding its taxonomy, the blast results obtained by using two molecular markers (ITS and TEF-1α) evidenced a high affinity (% of identity 99.8 and 100%, respectively) with *N. vitifusiforme.* As our phylogenetic analysis does not significantly support this result and also considering the complexity of the *Neofusicoccum* genus taxonomy, further investigation will consider the sequencing and analysis of the whole genome of our fungal strains. 

Fungal species of the *Botryosphaeriaceae* family are notoriously recognized as causal agents of serious plant diseases. The etiology of many of these diseases is not completely known [39] due to the complexity of the interaction between these fungi and forest plants, revealing a complex nature in which the infectious entity, the host, the environment, and their interactions with other microorganisms and organisms, such as harmful insects, may be equally important [19]. Regarding the diffusion of these fungi, it is well known that *Botryosphaeriaceae* species produce spores that are dispersed by wind, rain, and insects [19]. 

For example, some borer insects (i.e., *Coleoptera*: *Buprestidae*, *Curculionidae*, and *Cerambycidae*) such as the black-banded oak borer, *Coraebus florentinus,* considered an emerging oak tree pest in the Mediterranean region [40], as well as the oak pinhole borer, *Platypus cylindrus* [41], and the two wood-boring beetles *Cerambyx welensii* and *Coraebus fasciatus* [42], can play an important role. Nevertheless, the possible pathogenic role of endophytic fungi belonging to *Botryosphaeriaceae* has also been suggested [43]. One of the most accepted hypotheses about inoculum spreading suggests that spores are dispersed by wind and raindrops [44], although human movement and international trade seem to be the main cause of the long-distance dispersion of these fungi [45]. Due to the large number of potential hosts and the amount of plant material moving globally, it is nearly impossible to detect latent pathogens in asymptomatic material. Therefore, understanding the ecological niche requirements for potential species distribution areas may be a more effective way to predict and prevent future outbreaks.

## 5. Conclusions

In our investigation, we have identified and characterized some important fungal pathogens belonging to *Botryosphaeriaceae* and affecting holm oak forests in the woods of southern Italy. Understanding the ecological roles and interactions of these fungi, especially in conjunction with other stressors, is pivotal in carrying out control measures to reduce the impact of these pathogens on holm oak forests and also to preserve the landscape that has also been devastated by other serious plant pathogens (e.g., the quarantine bacterium *Xylella fastidiosa* in the Salento Peninsula, Apulia Region, Italy). Close attention should be paid to preventive measures to mitigate environmental stressors, harmful insects, bolstering forest resilience, and monitoring global trade to check the spread of new pathogens. Intervention strategies could include developing effective measures for early pathogen detection by also involving different molecular tools, such as PCR, q-PCR, and genomic and metagenomic analysis, to better identify and understand the role of plant endophytes. Furthermore, collaborative efforts among scientists, policymakers, and stakeholders are also crucial. Through shared knowledge and adaptive strategies, we can better understand disease dynamics and implement innovative approaches to safeguard the health and sustainability of oak forests. 

## Figures and Tables

**Table 1:** Geographic specifications of woodland involved in this study. All woodlands are located in the province of Lecce (Apulia, Italy).

Wood	Wood Name	Municipalities	Latitude (DD)	Longitude (DD)
1	Fraganite	Muro Leccese	40.099761	18.313251
2	Pozzo Mauro	Muro Leccese	40.098289	18.346773
3	Baia dei Turchi	Otranto	40.191555	18.463726
4	Maramonti	San Cassiano	40.052636	18.315900
5	Solicara	Lecce	40.435958	18.201963
6	Bosco Fiore	Lecce	40.385044	18.255363
7	Bosco San Biagio	Calimera	40.253310	18.303520

**Table 2:** GenBank accession numbers, hosts, and species identity for all *Botryosphaeriaceae* used in the phylogenetic analyses.

						Accession n. (GenBank)	
Genus	Species	Strain	Host	Country	Note	ITS	TEF-1 α
*Neofusicoccum*	*vitifusiforme*	QLE3	*Quercus ilex*	Italy	Isolated in this study	OQ876772	OQ750144
*Neofusicoccum*	*australe*	CBS119046	*Rubus* sp.	Portugal	/	DQ299244.1	EU017541.1
*Neofusicoccum*	*australe*	STE-U4425	*Vitis vinifera*	South Africa	/	AY343388.1	AY343347.1
*Neofusicoccum*	*luteum*	CBS110299	*Vitis vinifera*	Portugal	/	AY259091.1	AY573217.1
*Neofusicoccum*	*luteum*	CMW9076	*Malus x domestica*	New Zealand	/	AY339257.1	AY339265.1
*Neofusicoccum*	*vitifusiforme*	STE-U5252	*Vitis vinifera*	South Africa	/	AY343383.1	AY343343.1
*Neofusicoccum*	*vitifusiforme*	STE-U5050	*Vitis vinifera*	South Africa	/	AY343382.1	AY343344.1
*Neofusicoccum*	*viticlavatum*	STE-U5044	*Vitis vinifera*	South Africa	/	AY343381.1	AY343342.1
*Neofusicoccum*	*viticlavatum*	STE-U5041	*Vitis vinifera*	South Africa	/	AY343380.1	AY343341.1
*Neofusicoccum*	*parvum*	CBS110301	*Vitis vinifera*	Portugal	/	AY259098.2	AY573221.1
*Neofusicoccum*	*parvum*	CMW9081	*Populus nigra*	New Zealand	/	AY236943.1	AY236888.1
*Neofusicoccum*	*ribis*	96-187	*Ribes rubrum*	Not known	/	AF241177.1	AY236879.1
*Neofusicoccum*	*ribis*	CMW7772	*Ribes* sp.	USA	/	AY236935.1	AY236877.1
*Neofusicoccum*	*mangiferae*	CMW7024	*Mangifera indica*	Australia	/	AY615185.1	DQ093221.1
*Neofusicoccum*	*mangiferae*	CMW7797	*Mangifera indica*	Australia	/	AY615186.1	DQ093220.1
*Neofusicoccum*	*eucalyptorum*	CMW10125	*Eucalyptus grandis*	South Africa	/	AF283686.1	AY236891.1
*Neofusicoccum*	*eucalyptorum*	CMW10126	*Eucalyptus grandis*	South Africa	/	AF283687.1	AY236892.1
*Neofusicoccum*	*eucalypticola*	CMW6217	*Eucalyptus rossi*	Australia	/	AY615143.1	AY615135.1
*Neofusicoccum*	*eucalypticola*	CMW6539	*Eucalyptus grandis*	South Africa	/	AY615141.1	AY615133.1
*Lasiodiplodia*	*Mediterranea*	BL1	*Quercus ilex*	Italy	Outgroup Genera	KJ638312.1	KJ638331.1
*Diplodia*	*corticola*	QLE1	*Quercus ilex*	Italy	Isolated in this study	OQ876772	OQ750142
*Diplodia*	*quercivora*	QLE2	*Quercus ilex*	Italy	Isolated in this study	OQ831537	OQ750143
*Diplodia*	*corticola*	CBS112072	*Quercus ilex*	Spain	/	AY259108.1	JX894221.1
*Diplodia*	*quercivora*	BOT19	*Quercus suber*	Algeria	/	MF535381.1	MF535391.1
*Diplodia*	*corticola*	BL10	*Quercus ilex*	Italy	/	JX894191.1	JX894210.1
*Diplodia*	*quercivora*	BL8	*Quercus canariensis*	Tunisia	/	JX894205.1	JX894229.1
*Diplodia*	*scrobiculata*	BL5	*Arbutus unedo*	Italy	/	GU722102.1	JX894231.1
*Diplodia*	*seriata*	BL3	*Ulmus minor*	Italy	/	JX894207.1	JX894232.1
*Diplodia*	*mutila*	CBS112553	*Vitis vinifera*	Portugal	/	AY259093.2	AY573219.1
*Diplodia*	*mutila*	Sample301	*Juglans regia*	Chile	/	MW412902.1	MW574125.1
*Diplodia*	*sapinea*	CMW39341	*Cedrus deodara*	Montenegro	/	KF574998.1	KF575028.1
*Diplodia*	*sapinea*	CMW39338	*Cedrus atlantica*	Serbia	/	KF574999.1	KF575029.1
*Diplodia*	*seriata*	CBS112555	*Vitis vinifera*	Portugal	/	AY259094.2	AY573220.1
*Diplodia*	*seriata*	CBS119049	*Vitis* sp.	Italy	/	DQ458889.1	DQ458874.1
*Xylaria*	*hypoxylon*	GB6391	Isolated from soil	Mexico	Outgroup Order	AM993138.1	AY327490.1

## Data Availability

The data underlying this article will be shared on reasonable request to the corresponding author.
